# Correlation between diameter of esophageal varices and early rebleeding following endoscopic variceal ligation: a multicenter retrospective study based on artificial intelligence-based endoscopic virtual rule

**DOI:** 10.3389/fmed.2024.1406108

**Published:** 2024-06-12

**Authors:** Chuankun Cao, Jing Jin, Rui Cai, Yuan Chu, Kai Wu, Zuo Wang, Ting Xiao, Heng Zhang, Hongfei Huang, Heng Liu, Qianqian Zhang, Xuecan Mei, Derun Kong

**Affiliations:** ^1^Department of Gastroenterology, The First Affiliated Hospital of Anhui Medical University, Anhui Provincial Key Laboratory of Digestive Diseases, Hefei, China; ^2^Department of Gastroenterology, Phoenix Hospital of Huainan Oriental Hospital Group, Huainan, China; ^3^Department of Gastroenterology, General Hospital of Huainan Oriental Hospital Group, Huainan, China

**Keywords:** liver cirrhosis, esophageal varices, endoscopic variceal ligation, early rebleeding, post-ligation ulcer, artificial intelligence, portal hypertension

## Abstract

**Background and objective:**

Bleeding following endoscopic variceal ligation (EVL) may occur as a result of numerous factors, including a diameter of esophageal varices (EV) that is too large to be completely ligated. The present study aimed to develop an artificial intelligence-based endoscopic virtual ruler (EVR) to measure the diameter of EV with a view to finding more suitable cases for EVL.

**Methods:**

The present study was a multicenter retrospective study that included a total of 1,062 EVLs in 727 patients with liver cirrhosis with EV, who underwent EVL from April 2016 to March 2023. Patients were divided into early rebleeding (*n* = 80) and non-rebleeding groups (*n* = 982) according to whether postoperative bleeding occurred at 6 weeks. The characteristics of patient baseline data, the status of rebleeding at 6 weeks after surgery and the survival status at 6 weeks after rebleeding were analyzed.

**Results:**

The early rebleeding rate following 1,062 EVL procedures was 7.5%, and the mortality rate at 6 weeks after bleeding was 16.5%. Results of the one-way binary logistic regression analysis demonstrated that the risk factors for early rebleeding following EVL included: high TB (*P* = 0.009), low Alb (*P* = 0.001), high PT (*P* = 0.004), PVT (*P* = 0.026), HCC (*P* = 0.018), high Child-Pugh score (*P* < 0.001), Child-Pugh grade C(*P* < 0.001), high MELD score(*P* = 0.004), Japanese variceal grade F3 (*P* < 0.001), diameter of EV (*P* < 0.001), and number of ligature rings (*P* = 0.029). Results of the multifactorial binary logistic regression analysis demonstrated that Child-Pugh grade C (*P* = 0.007), Japanese variceal grade F3 (*P* = 0.009), and diameter of EV (*P* < 0.001) may exhibit potential in predicting early rebleeding following EVL. ROC analysis demonstrated that the area under curve (AUC) for EV diameter was 0.848, and the AUC for Japanese variceal grade was 0.635, which was statistically significant (*P* < 0.001). Thus, results of the present study demonstrated that EV diameter was more optimal in predicting early rebleeding following EVL than Japanese variceal grade criteria. The cut-off value of EV diameter was calculated to be 1.35 cm (sensitivity, 70.0%; specificity, 89.2%).

**Conclusion:**

If the diameter of EV is ≥1.4 cm, there may be a high risk of early rebleeding following EVL surgery; thus, we recommend caution with EVL.

## 1 Introduction

Esophagogastric variceal bleeding (EVB) is one of the most serious and aggressive complications of portal hypertension in liver cirrhosis, and is the leading cause of death in patients with cirrhosis (1). Esophagogastric varices are present in ~52% of patients with cirrhosis, and varices are present in 50–60% of patients with compensated cirrhosis and up to 85% of patients with decompensated cirrhosis (2). The rate of variceal bleeding is 5–15% per year (3), and the mortality rate after 6 weeks of variceal bleeding is as high as 15–25% (4).

At present, EVL is a first-line treatment method for preventing first EVB (primary prophylaxis), controlling acute esophageal-gastric variceal bleeding, and preventing second EVB (secondary prophylaxis). Notably, EVL is recommended by numerous national and regional gastroenterology and hepatology society guidelines, including those in Europe and America (1, 5–8). Early postoperative rebleeding is a common adverse event associated with EVL, and occurs in 4.8–15.6% of patients following EVL (9–12). Moreover, early postoperative rebleeding may be heavy and uncontrollable, with mortality rates as high as 26.9–38.3% (10–12).

Early rebleeding following EVL is influenced by numerous factors, including an esophageal varices (EV) diameter that is too large to be completely ligated. For complete ligation, and to ensure that the surface mucosa of the target vessel, including the proximal and contralateral walls of the vessel, are completely absorbed into the ligator, EVL is only used for varices of a medium diameter. Moreover, the ligation ring may detach following EVL and fatal rebleeding may occur. Thus, the Chinese Guidelines for Liver Cirrhosis (8) state that an EV diameter of >2.0 cm is a contraindication for EVL, and results of a previous study suggested that an EV diameter >1.0 cm should be included as a contraindication for EVL (13). Notably, the measurement methods for determining the diameter of EV are suboptimal, and there is no reference available in the esophageal lumen. At present, visual assessments are most commonly used for estimating EV diameter; however, these are based on the subjective judgment of endoscopists, and the results may vary depending on prior experience (14).

Selecting different endoscopic treatments according to the diameter of varices aids in the treatment of esophagogastric varices. Thus, a non-invasive measurement technique was developed using endoscopic artificial intelligence (AI); namely, an endoscopic virtual ruler (EVR, Hefei Zhongna Medical Instrument Co., Ltd, Hefei, China) (15), to measure EV diameter without contact. The present study aimed to determine the potential association between the diameter of EV and early rebleeding following EVL, to determine the selection criteria for the appropriate diameter of EV for EVL.

## 2 Materials and methods

### 2.1 Clinical data

The present multicenter retrospective cohort study included 727 patients with cirrhosis with EV who underwent EVL from April 2016 to March 2023 in The First Affiliated Hospital of Anhui Medical University, General Hospital of Huainan Oriental Hospital Group and Phoenix Hospital of Huainan Oriental Hospital Group. There was a total of 1,062 EVLs.

Videos and images were available for all cases. During gastroscopy, a transparent cap with an inner diameter of 1 cm (cat. no. DL-108-40; Micro Tech Co., Ltd., Nanjing, China) was installed at the front end of the endoscope to detect the vessel diameter.

**Figure 1:**
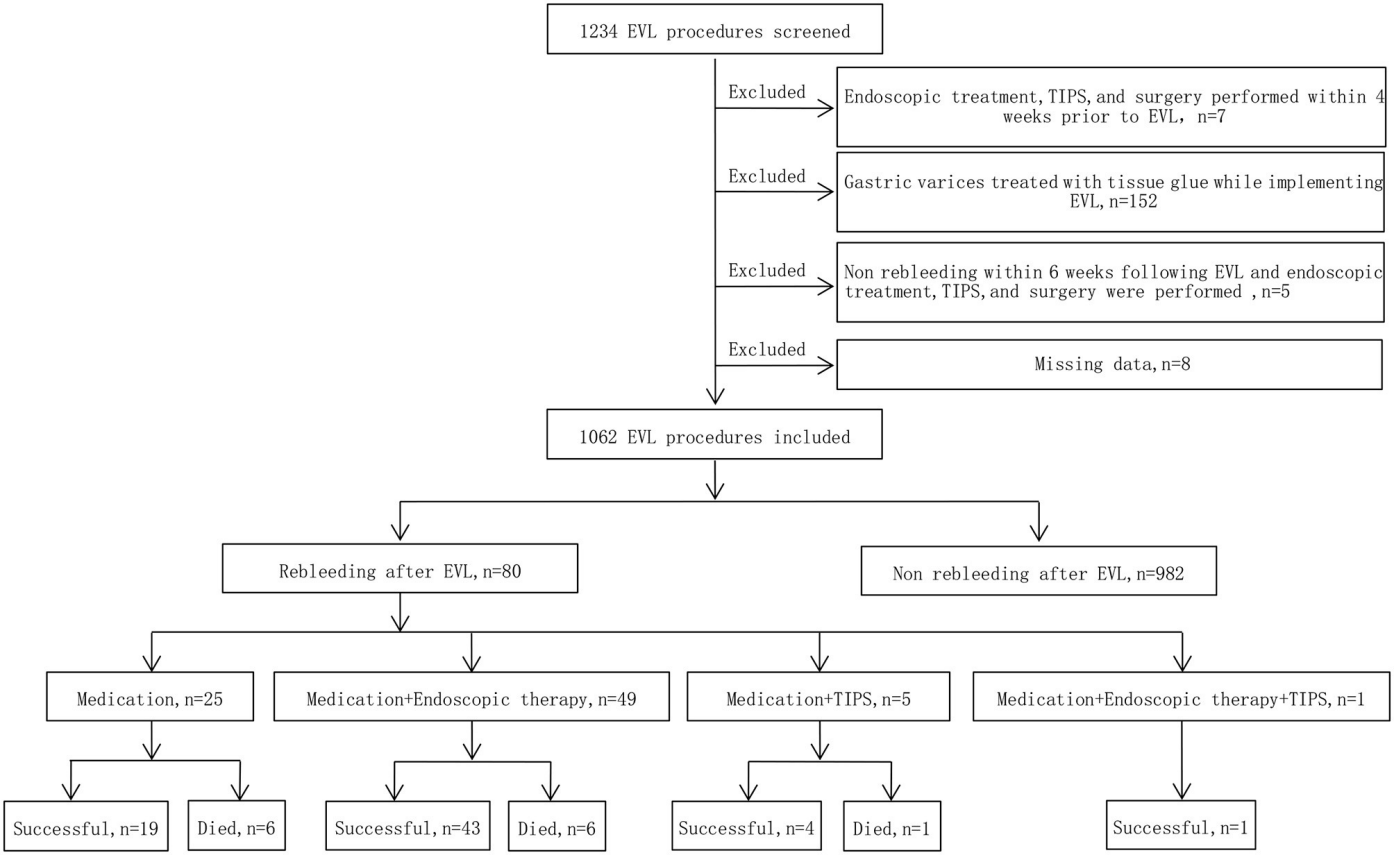
Study flow chart. EVL, endoscopic variceal ligation; TIPS, transjugular intrahepatic portosystemic shunt.

The exclusion criteria were as follows: ① Endoscopic treatment of varices, transjugular intrahepatic portosystemic shunt (TIPS), surgical shunts or devascularization surgery within 4 weeks prior to EVL; ② gastric varices treated with tissue glue while implementing EVL; ③ no rebleeding within 6 weeks following EVL and endoscopic treatment of varices, TIPS, surgical shunts or devascularization surgery were performed; ④ patients with active bleeding:Under endoscopy esophageal varices are spurting blood, or oozing so much blood that the field of view is so unclear that it is impossible to apply EVR to accurately measure EV diameter; and ⑤ missing data. The detailed study flow is displayed in Figure 1.

Endoscopic and medical records were reviewed to obtain demographic, clinical, endoscopic and follow-up data. Analyses were conducted to determine the presence of rebleeding at 6 weeks following EVL and death at 6 weeks after bleeding.

The present study was conducted in accordance with the Declaration of Helsinki and Good Clinical Practice Guidelines, and was approved by the Ethics Committee of the First Affiliated Hospital of Anhui Medical University (ethics approval no. PJ2022-10-16). The present study was registered as a clinical trial, with the Chinese Clinical Trial Registry Registration number (trial ID): ChiCTR2200064028(the date of first registration: 24/09/2022). Written consent was obtained from all patients for the endoscopic procedure. Due to the retrospective nature of the present study, the anonymity of data, and the routine nature of all clinical procedures and tests conducted, the requirement for specific informed consent was waived.

### 2.2 Severity grade of EV

Two grade models were used for the present study:

① Classical morphological assessment

Three physicians with 10 years of endoscopic experience who had carried out >100 EVL cases reviewed the endoscopic features, videos and images of all cases with varices, and graded the varices. According to the guidelines of the Japan Society for Portal Hypertension (16), the grading criteria of EV (referred to as the Japanese variceal grade) are as follows: F1, straight and small-calibered varices; F2, moderately enlarged, beady varices occupying less than one third of the esophageal lumen; and F3, markedly enlarged, nodular or tumor-shaped varices occupying more than one third of the esophageal lumen.

② EVR guides the evaluation of EV diameter

**Figure 2:**
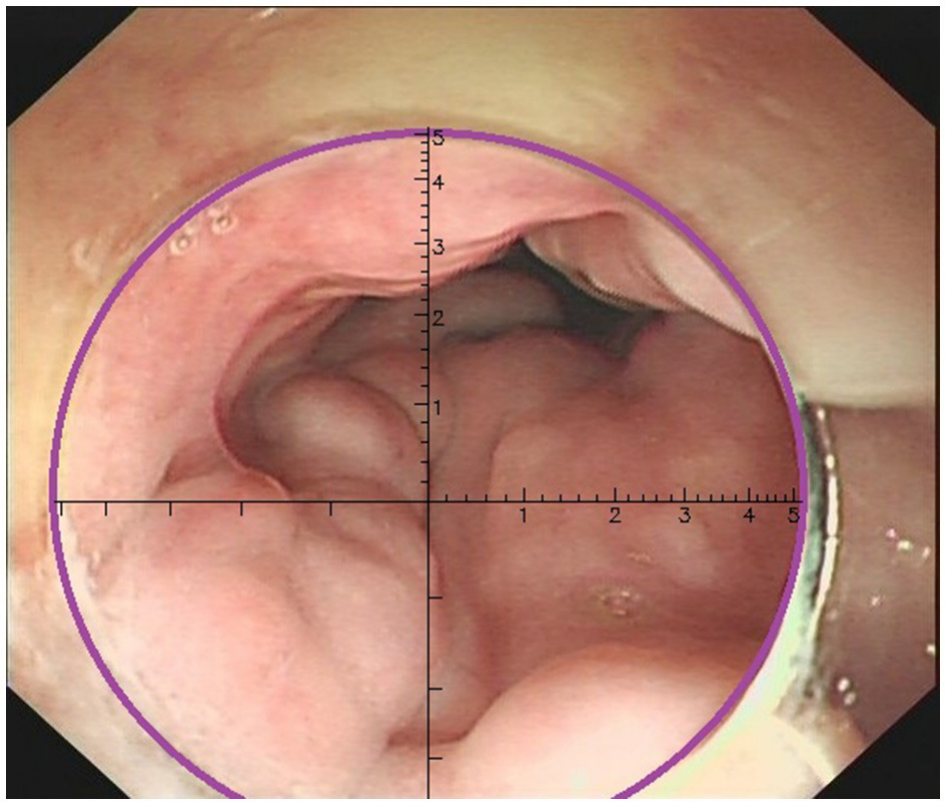
The end of the transparent cap was marked as a discontinuous arc in the endoscopic field of view (purple arc on the figure). When the artificial intelligence recognized the arc, it automatically formed a coordinate system.

**Figure 3:**
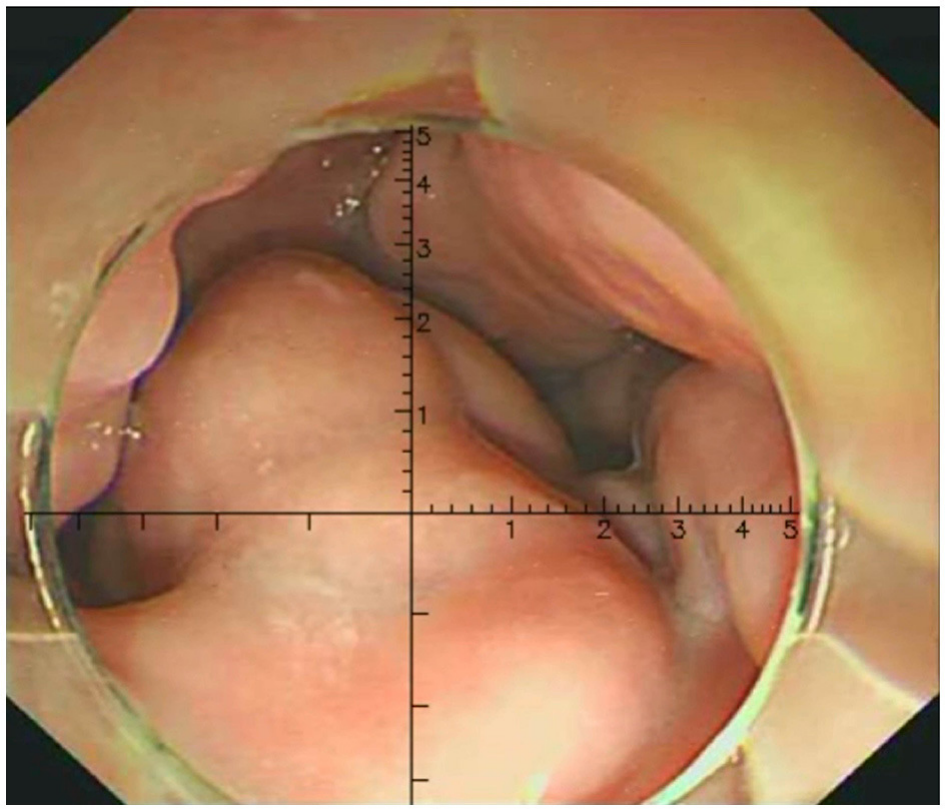
Measurement of the EV diameter using EVR by images. The variceal diameter of the patient was about 0.6 cm.

Three physicians, each with over 10 years of endoscopic experience and having performed more than 100 EVL cases, conducted a comprehensive review of the endoscopic features, videos, and images of all cases. They made meticulous observations and precise measurements using EVR. The endoscopic videos and images of all patients were incorporated into the EVR to measure and record the diameter of the largest vessel. EVR is an AI-assisted technique that connects a transparent cap with an inner diameter of 1 cm to the front end of the endoscope to use as a reference. EVR is constructed using algorithms, such as Gaussian filters, Canney Edge detector and Hove circles. Notably, EVR was corrected using a barrel deformation experiment. When approaching the target EV, the discontinuous arc of the cap was detected using AI, and a Cartesian coordinate system was subsequently established in the center of a circle corresponding to the arc (Figure 2). The diameter of EV was determined using the aforementioned coordinate system, and EV size was obtained through reading the scale on a ruler. Retrospective data analysis was performed using EVR software to detect the diameter of EV in endoscopic videos or images. Notably, each endoscopy video used was the original, with a tip cap at the front end of the endoscope. For EV with a diameter >1 cm, the secondary mode of EVR was used. The distance between the anterior cap of the endoscope and the blood vessel was evaluated using the multiplication method to obtain the EV diameter, and this was calculated according to a 1:2 ratio, where a diameter of 2 cm is displayed as 1 cm in EVR. One case of EVR measurement of the variceal diameter by images is displayed in Figure 3. One case of EVR measurement of the variceal diameter by videos is displayed in Supplementary Video 1.

### 2.3 EVL techniques, main research indicators, and associated concept definitions

EVL was performed in the three hospitals according to standard procedures (17). All EVLs were performed by experienced gastroenterologists with 10 years of endoscopic experience who had carried out >100 EVL cases. The endoscope used exhibited a tip diameter of ~9.9 mm (cat. no. GIF-Q260J; Olympus Corporation, Japan). The Saeed Six Shooter ligature device was used (cat. no. MBL-6-F; Wilson Cook Medical Incorporated, Winston Salem, NC, USA). Vasoactive drugs (Octreotide or Somatostatin) were uses for 3 days after EVL in all patients with emergency EVLs.

The primary indicator of the present study was postoperative rebleeding at 6 weeks. The secondary indicator of the present study was all-cause mortality at 6 weeks following bleeding, including death due to upper gastrointestinal bleeding, liver failure, hepatic encephalopathy, hepatorenal syndrome or infection. According to the Baveno II (18) standard, any cause of death within 6 weeks of a bleeding episode is considered a bleeding-related death.

Early rebleeding following EVL was defined as recurrence of active bleeding within 6 weeks following EVL. Symptoms of early rebleeding included vomiting blood, black stools or blood in stools; a systolic blood pressure decrease of >20 mmHg or a heart rate increase of >20 beats/min; or a hemoglobin decrease of >30 g/l in the absence of a blood transfusion.

Post-ligation ulcer hemorrhage included postoperative bleeding originating from a post-EVL esophageal ulcer, with an appearance consistent with a banded ulcer and the presence of blood secretion; or a visible blood clot, a pigmented base or blood in the stomach in the absence of alternative sources of upper gastrointestinal bleeding.

### 2.4 Statistical analysis

Statistical analyses were performed using SPSS (version 26.0; IBMCorp., Armonk, NY, USA). Quantitative data are expressed as the mean ± standard deviation (x ± s), and qualitative data are expressed as frequencies and percentages. Consistency within and between groups was analyzed using intraclass correlation efficient (ICC) for physician visual assessment and EVR measurement of esophageal variceal diameter. The potential association between Japanese variceal grade and esophageal variceal diameter measured using EVR with early rebleeding following EVL was determined using one-way binary logistic regression. The predictive ability of either Japanese variceal grade or esophageal variceal diameter on early rebleeding following EVL was further assessed using receiver operating characteristic (ROC) curves. AUC >0.6 indicated an average level of accuracy, and AUC >0.8 indicated a high level of accuracy. *P* < 0.05 was considered to indicate a statistically significant difference.

## 3 Results

### 3.1 Basic patient characteristics

A total of 727 patients were included in the present study, including 498 males and 229 females. Notably, there were 410 cases of liver cirrhosis caused by hepatitis B, 15 cases of liver cirrhosis caused by hepatitis C, 67 cases of alcoholic cirrhosis, 33 cases of hepatitis B and alcohol, 4 cases of hepatitis C and alcohol, 65 cases of autoimmune disease, 11 cases of schistosomiasis, and 122 cases with unknown causes. A total of 1,062 EVL procedures were performed. The baseline indicators of patients are displayed in Table 1.

**Table 1:** Characteristics of all patients with 1,062 EVLs included in the present study.

**Characteristic**	**Value**
Age, years	55.0 ± 11.6
HGB, g/L	87.4 ± 27.5
PLT, /L	(86.9 ± 75.3) × 10^9^
ALT, U/L	40.7 ± 135.1
TB, umol/L	25.0 ± 19.9
Alb, g/L	34.1 ± 5.4
Creatinine, umol/L	66.2 ± 36.7
Blood Na, mmol/L	139.3 ± 3.7
Fasting blood sugar, mmol/L	6.4 ± 2.6
PT, s	15.5 ± 2.7
INR	1.3 ± 0.5
PVT, *n* (%)	204 (19.2%)
HCC, *n* (%)	114 (10.7%)
HE, *n* (%)	20 (1.9%)
Child-Pugh score	7.0 ± 1.7
**Child-Pugh grade**	
Child A grade, *n* (%)	486 (45.8%)
Child B grade, *n* (%)	478 (45.0%)
Child C grade, *n* (%)	98 (9.2%)
Meld score	9.0 ± 4.1
First EVL, *n* (%)	727 (68.5%)
Not first EVL, *n* (%)	335 (31.5%)
**Indications for surgery**	
Emergent EVL, *n* (%)	145 (13.7%)
Primary prophylaxis, *n* (%)	139 (13.1%)
Secondary prophylaxis, *n* (%)	778 (73.3%)
**Postoperative medication**	
Carvedilol, *n* (%)	610 (57.4%)
Propranolol, *n* (%)	43 (4.0%)
None of the above drugs, *n* (%)	409 (38.5%)
Vasoactive drugs, *n* (%)	145 (13.7%)
**Vascular fractionation**	
EV, *n* (%)	834 (78.5%)
GOV1, *n* (%)	187 (17.6%)
GOV2, *n* (%)	41 (3.9%)
**Location**	
Upper middle and lower section, *n* (%)	185 (17.4%)
Lower middle section, *n* (%)	591 (55.6%)
Lower section, *n* (%)	286 (26.9%)
RC signs, *n* (%)	952 (89.6%)
**Size of varices**	
F1 grade, *n* (%)	64 (6.0%)
F2 grade, *n* (%)	331 (31.2%)
F3 grade, *n* (%)	667 (62.8%)
Diameter of EV, cm	1.0 ± 0.3
Number of ligature rings, n	6.7 ± 2.2

PVT, portal vein thrombosis; HCC, hepatocellular carcinoma; HE, hepatic encephalopathy; MELD, model for end-stage liver disease; EVL, endoscopic variceal ligation; EV, esophageal varices; GOV, gastroesophageal varice; RC, red color.

### 3.2 Distribution of esophageal variceal diameter

**Figure 4:**
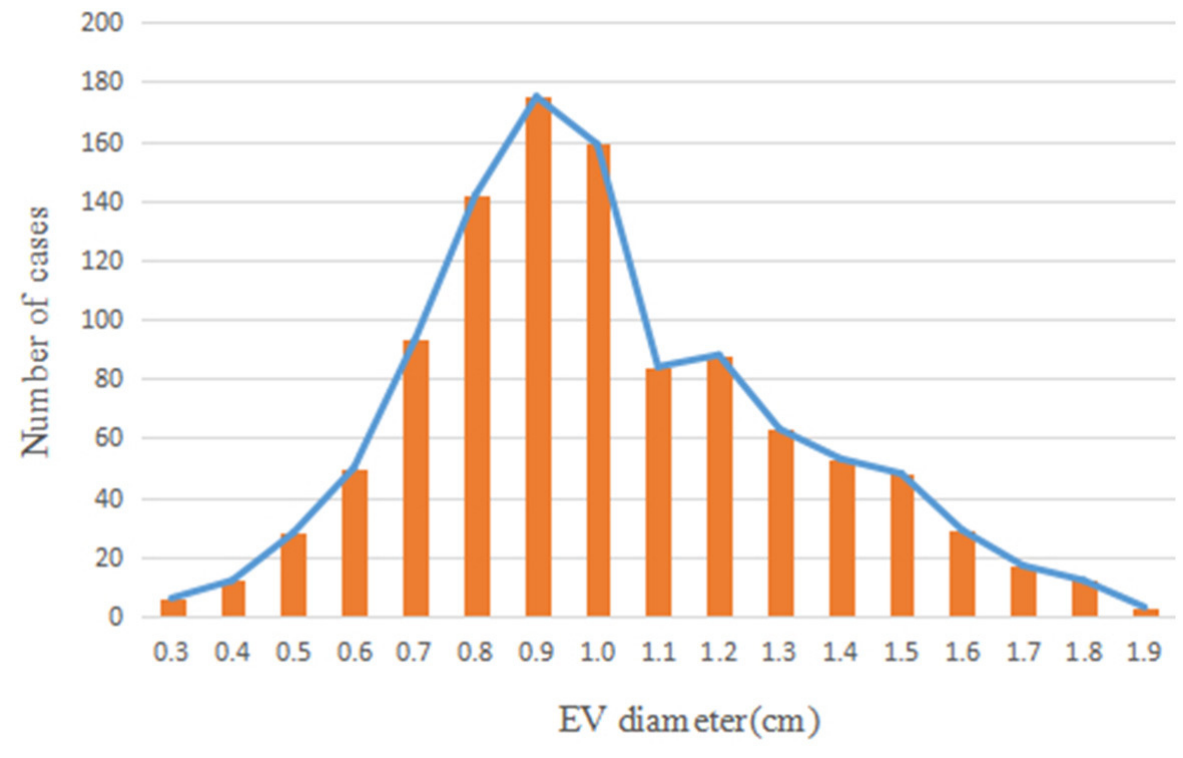
Distribution map of EV diameters. EV, esophageal varices.

Patients included in the present study exhibited an approximate normal distribution, with the majority of cases exhibiting a peak EV diameter of 0.9 cm. The distribution of EV diameters is displayed in Figure 4.

### 3.3 Consistency within and between two groups

An analysis using intraclass correlation efficient (ICC) was performed and found that the intraclass correlation efficient between physician visual assessment and EVR measurement of EV diameters was 0.815, and that the intraclass correlation efficient among the three physicians' visual assessment of EV diameters was 0.870, and the intraclass correlation efficient among the three physicians' measurements of EV diameters using the EVR was 0.965.

### 3.4 Early postoperative rebleeding rate and mortality

There were 80 cases of early postoperative rebleeding at 6 weeks with a bleeding rate of 7.5% (80/1,062), including 1 case of postoperative bleeding in grade F1 patients, 9 cases of postoperative bleeding in grade F2 patients and 70 cases of postoperative bleeding in grade F3 patients. The bleeding rate was 11.0% (16/145 patients) following emergency EVLs, while the bleeding rate observed following prophylactic EVLs was 7.0% (64/917). The postoperative bleeding rate following emergency EVLs was higher than that of prophylactic EVLs; however, the difference was not statistically significant (*P* = 0.088).

Despite additional medication, endoscopic treatments or TIPS, 13 patients with postoperative bleeding died in the present study. Among 80 cases of bleeding following EVL, 1 patient experienced two early bleeding episodes following EVL. The 6-week mortality rate following a bleeding episode was 16.5% (13/79 patients). In the present study, numerous patients did not undergo endoscopy and treatment due to effective medication or unstable vital signs due to heavy bleeding. Among 50 patients with postoperative bleeding who underwent endoscopic diagnosis and treatment, 42 cases of bleeding originated from ulcers following ligation, and 8 cases of bleeding originated from ruptured EV that were not occluded.

### 3.5 Predictive potential of EV diameter for early rebleeding following EVL

In a one-way binary logistic regression analysis of the patients' relevant data, various factors were examined to determine their association with early rebleeding following EVL. The analysis revealed several significant risk factors. These included high total bilirubin (TB) levels (*P* = 0.009), low albumin (Alb) levels (*P* = 0.001), elevated prothrombin time (PT) (*P* = 0.004), presence of portal vein thrombosis (PVT) (*P* = 0.026), hepatocellular carcinoma (HCC) (*P* = 0.018), high Child-Pugh score (*P* < 0.001), Child-Pugh grade C (*P* < 0.001), high Model for End-Stage Liver Disease (MELD) score (*P* = 0.004), Japanese variceal grade F3 (*P* < 0.001), diameter of EV (*P* < 0.001), and number of ligature rings used (*P* = 0.029) (Table 2). These findings underscore the importance of considering these factors in the management and risk assessment of patients undergoing EVL.

**Table 2:** One-way binary logistic regression analysis of risk factors for early rebleeding following EVL.

**Variable**		**Non-rebleeding (*n* = 982)**	**Rebleeding (*n* = 80)**	***P***	**OR**
Sex	Female, *n*	310	21		
	Male, *n*	672	59	0.325	0.772
Age, years		55.0 ± 11.5	56.0 ± 12.2	0.445	1.008
Causes of cirrhosis	Viral hepatitis, *n*	566	42		
	Acohol, *n*	100	3	0.136	0.404
	Viral hepatitis+alcohol, *n*	55	7	0.212	1.715
	Autoimmune, *n*	92	10	0.301	1.465
	Other reasons, *n*	169	18	0.221	1.435
HGB, g/L		87.8 ± 27.5	82.5 ± 27.2	0.091	0.992
PLT,/L		(87.0 ± 76.4) × 10^9^	(85.8 ± 60.5) × 10^9^	0.888	1
ALT, U/L		40.8 ± 140.1	38.5 ± 38.2	0.882	1
TB, umol/L		24.5 ± 19.8	30.8 ± 20.5	0.009	1.011
Alb, g/L		34.2 ± 5.4	32.2 ± 5.6	0.001	0.934
Creatinine, umol/L		66.4 ± 36.8	64.0 ± 35.9	0.582	0.998
Blood Na, mmol/L		139.3 ± 3.8	139.2 ± 3.4	0.914	0.997
Fasting blood sugar, mmol/L		6.4 ± 2.6	6.6 ± 2.1	0.360	1.038
PT, s		15.4 ± 2.6	16.3 ± 3.6	0.004	1.099
INR		1.3 ± 0.5	1.3 ± 0.2	0.415	1.143
PVT, *n*		181	23	0.026	1.786
HCC, *n*		99	15	0.018	2.058
Large amount of ascites, *n*		16	3	0.182	0.425
None+small+moderate amount of ascites, *n*		966	77		
HE, n		17	3	0.213	2.212
Child-Pugh score		6.9 ± 1.7	8.0 ± 2.0	0	1.360
Child-Pugh grade C, *n*		71	27	0	6.537
Child-Pugh grade A+B, *n*		911	53		
Meld score		8.9 ± 4.1	10.3 ± 4.0	0.004	1.077
Not first EVL, *n*		309	26	0.848	1.049
First EVL, *n*		673	54		
Indications for surgery	Emergent EVL, *n*	129	16	0.088	1.653
	Prophylactic EVL, *n*	853	64		
Postoperative medication	Carvedilol, *n*	567	43	0.749	0.925
	Propranolol, *n*	37	6	0.154	1.977
	None, *n*	378	31		
Vasoactive agents, *n*		129	16	0.088	1.653
Vascular fractionation	EV, *n*	774	60	0.674	
	GOV1, *n*	170	17	0.376	1.290
	GOV2, *n*	38	3	0.976	1.018
Location	Upper middle and lower section, *n*	166	19		
	Lower middle section, *n*	547	44	0.221	0.703
	Lower section, *n*	269	17	0.088	0.552
RC signs, *n*		879	73	0.624	1.222
Japanese variceal grade	F3, *n*	597	70	0	4.514
	F1+F2, *n*	385	10		
Diameter of EV, cm		1 ± 0.3	1.4 ± 0.3	0	171.186
Number of ligature rings, *n*		6.7 ± 2.1	7.2 ± 2.8	0.029	1.102

EV, esophageal varices; EVL, endoscopic variceal ligation; OR, odds ratio.

Variables with *P* < 0.05 in one-way logistic regression analysis further selected: high TB, low Alb, PVT, HCC, high Child-Pugh score, Child-Pugh grade C, high MELD score, Japanese variceal grade F3, diameter of EV, and number of ligature rings were included in multifactorial binary logistic regression analysis. The results showed that the independent influences of early rebleeding following EVL were Child-Pugh grade C (*P* = 0.007), Japanese variceal grade F3 (*P* = 0.009), and diameter of EV (*P* < 0.001) which may exhibit potential in predicting early rebleeding following EVL.

**Figure 5:**
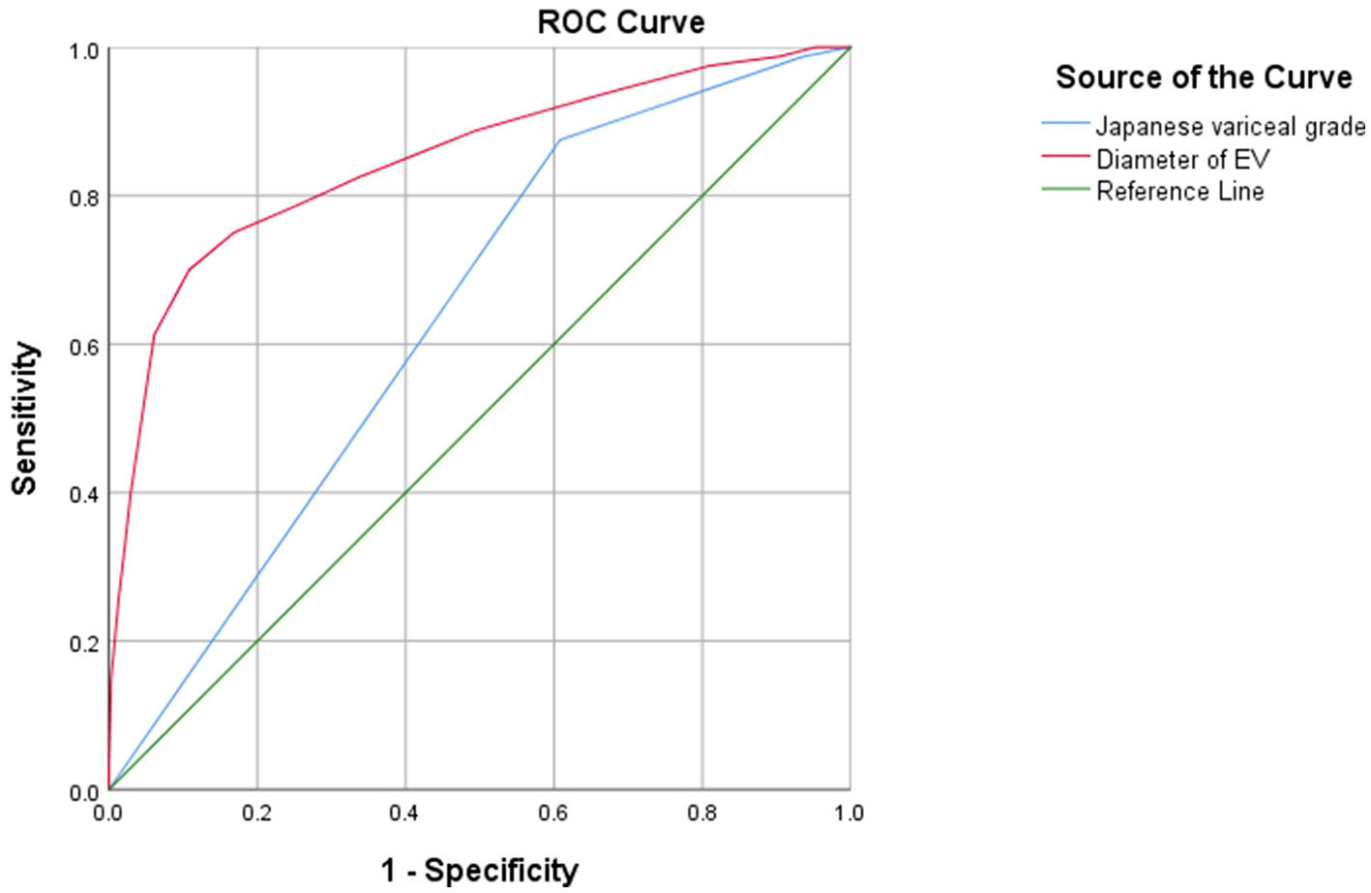
EV diameter and Japanese variceal grade as predictive factors for early rebleeding following EVL.

Results of the ROC analysis revealed that the EV diameter AUC was 0.848 (95% CI, 0.797–0.899; *P* < 0.001), and the Japanese Variceal grade AUC was 0.635 (95% CI, 0.580–0.690; *P* < 0.001), as shown in Figure 5. Results of further statistical analysis demonstrated that the predictive value of EV diameter for early postoperative rebleeding was greater than that of Japanese Variceal grade [z = 10.991; Sig. (2-tail)^a^ = 0; AUC difference, 0.213; standard error (SE) difference^b^ = 0.213, asymptotic 95% CI, 0.175–0.251]. EV diameter is a continuous variable, and the cut-off value was determined as 1.35 cm, with a sensitivity of 70.0% and a specificity of 89.2%. In the present study, the relatively low sensitivity may be associated with the lower early postoperative rebleeding rate of 7.5%.

## 4 Discussion

Stiegmann et al. (19) initially carried out EVL in the treatment of esophageal variceal bleeding in 1986. Subsequently, EVL was compared with endoscopic injection sclerotherapy (EIS), and results demonstrated that adverse events were reduced at a higher level following EVL; thus improving survival, eliminating varices and reducing rebleeding rates (20, 21). Therefore, EVL is selected as the preferred option for the endoscopic treatment of EV. However, EVL may exert potentially fatal adverse events, such as early rebleeding following EVL. Results of the present study demonstrated that the rebleeding rate at 6 weeks following EVL was 7.5%, and the mortality rate at 6 weeks following bleeding was 16.5%. Results obtained by Sarin et al. (22) highlighted that bleeding following EVL may be a result of post-ligation ulcers, ruptured unoccluded EV, bleeding from ruptured gastric varices or portal hypertensive gastropathy. Notably, results of previous studies demonstrated that ulcers were not the main source of early rebleeding following EVL (10); however, numerous studies demonstrated the opposite results (12, 23, 24). Thus, in recent years, research is focused on adverse events following EVL, such as post-ligation ulcer hemorrhage. Notably, EVL is a standardized method that pre-sets the volume of varices that are absorbed into the cylinder for ligation. EVL is limited by the capacity of the ligation device; thus, only ligating the mucosa and submucosa (25). Shallow ulcers and inflammatory infiltration above the superficial submucosa may form once the ligated tissues at the EVL treatment site are shed. Notably, the ulcers are round with a white fibrin base, with a diameter of 10–12 mm and a depth of 1–2 mm. Ulcers often heal within 2–3 weeks (26). All EVLs result in postoperative ulcers; however, postoperative bleeding does not occur following every EVL. A high severity of liver disease and high blood sugar may result in the poor healing of ulcers; thus, postoperative bleeding (11, 27, 28). Our study found that Child-Pugh grade C, Japanese variceal grade F3, and diameter of EV may exhibit potential in predicting early rebleeding following EVL. On the other hand, postoperative bleeding may only occur when EV is too large for the vessels to be completely ligated, leading to ligature ring detachment and exposure of broken vessels below the ulcer (9). The Chinese Guidelines for Liver Cirrhosis (8) indicate that EV with a diameter >2.0 cm is not suitable for EVL. However, the diameter of blood vessels is judged by independent examiners and is therefore subjective, and the exact diameter may not be accurately stated. Therefore, the misjudgment of endoscopists may lead to inappropriate ligation and bleeding events following EVL.

At present, research into the impact of EV diameter on early bleeding following EVL is limited. Results of the present study revealed a high risk of early rebleeding following EVL in patients with EV Japanese variceal grade F3 and a large EV diameter. Despite additional medication, endoscopic treatment and TIPS following bleeding, 13 people died in the present study. Notably, the 6-week mortality rate following bleeding was 16.5%. In addition, there were 667 cases of grade F3 varices, with an early postoperative rebleeding rate of 10.5% (70/667). Thus, not all grade F3 EVs were suitable for EVL. EVR was subsequently used to measure the diameter of EV, and further statistical analysis revealed a cut-off value of 1.35 cm. Results of the present study demonstrated that the probability of early bleeding following EVL in patients with an EV diameter ≥1.4 cm was 34.6% (56/162). Considering the differences in ligation levels and the potential errors in assessment of variceal diameters, we recommend caution with EVL if the EV diameter is ≥1.4 cm. The percentage of patients with an EV diameter of ≥1.4 cm was relatively small, accounting for 15.3% (162/1,062) of patients included in the present study.

During successful EVLs, the static inner diameter and retraction force of the rubber band are equal, and the early detachment of the ligation ring is associated with the size of the EV. Prior to the formation of variceal thrombosis, if the rubber band detaches prematurely, the ligation site histologically demonstrates mucosal necrosis, vascular necrosis and the continuous dilation of varices; thus, the risk of bleeding remains high (29). Chen et al. (30) reported 7 cases of hemorrhage from 15 h to 9 days following EVL, which were considered to be associated with large varices, with a diameter exceeding the maximum diameter that should be ligated. During ligation, not all varices are absorbed into the cylinder of the ligature; thus, the entire vein is not ligated and blood flow is not completely blocked. This may lead to a lack of secondary thrombosis, slipping of the ligature ring and hemorrhage. Thus, EVL requires complete ligation. Ligation of the mucosa and the entire blood vessel, including the contralateral blood vessel wall, is required to achieve complete ligation effects and prevent the ligation ring from detaching. At present, the six-ring ligation device is widely used in clinical practice, with a diameter of ~10 mm and a front cylinder length of ~10 mm. Notably, the ligation volume depends on the corresponding cylinder, not on the elasticity of the band (31). Therefore, the maximum diameter of the varices that can be ligated by the ligation device is limited to a certain range. Li et al. (13) demonstrated that the diameter of the porcine varices impacted the degree of complete ligation *in vitro*, and the larger the diameter, the lower the proportion of complete ligation. Results of the present study demonstrated that large varices which exceed the maximum diameter for ligation may lead to early detachment of the ligature ring; thus, impacting healing of the ulcer, leading to heavy bleeding. Therefore, EVs that are too large are not suitable for ligation.

The most important factor for the prediction of variceal bleeding in cirrhotic portal hypertension is the size of the varices, and the risk of bleeding in patients with large varices is as high as 17% per year (32). The American Association for Liver Research recommends using a two-level classification system to determine the size of EV, and proposed a 5-mm threshold to classify varices into small and large groups. Thus, treatment and follow-up strategies are selected in line with varices size. Notably, accurate measurement of EV diameter, and the correct classification of large and small veins is required, for selecting treatment strategies and monitoring follow-up. A non-invasive measurement technique with endoscopic AI; namely, EVR, was used in the present study. In this study, consistency within and between groups was analyzed using intraclass‘g correlation efficient (ICC) for physician visual assessment and EVR measurement of esophageal variceal diameter. The results showed that the intraclass correlation efficient for measuring esophageal variceal diameters using EVR were higher than that for visual assessment, suggesting that EVR measurements are highly consistent and more reliable. Notably, EVR exhibits multiple advantages, including increased levels of accuracy in detecting and measuring EV diameter, as demonstrated in a previous study (15). In addition, EVR is user-friendly, as additional equipment and instruments are not required. EVR is cost-effective, time-efficient and does not impose additional burden or risk on the patient. Moreover, as a virtual measurement tool, EVR may be used to retrospectively measure the diameter of varices, as well as measuring in real-time during endoscopic examination. EVR is useful software for the endoscopic detection and treatment of EV, and allows endoscopists to objectively measure the variceal diameter; thus, assessing the risk of bleeding. In addition, EVR may provide an important reference for selecting an appropriate treatment strategy.

Although the present study is a multicenter and large-scale study, there are numerous limitations. For example, follow-up was limited in some patients due to the retrospective nature of the present study. In addition, the distance between the front cap of the endoscope and the varices is uncontrollable, and the transparent cap of the endoscope must be placed close to the target vessel. Moreover, not all endoscopists use a transparent cap in experimental procedures, and EVR detection cannot be carried out without a transparent cap. Therefore, improvements in EVR technology and further studies with larger sample sizes are required.

In conclusion, post-ligation ulcer hemorrhage is an adverse event associated with EVL, and the associated mortality rate is high. We recommend caution with EVL if the EV diameter is ≥1.4 cm because of the high risk of early rebleeding following EVL surgery, and alternative treatments, such as non-selective beta blockers, TIPS and EIS maybe should be considered. Notably, the technology of EIS is inconsistent, which may impact the potential therapeutic effects. The development of novel instruments and technologies may significantly improve the efficacy of EIS (33); however, further investigations are required.

## Data availability statement

The datasets presented in this study can be found in online repositories. The names of the repository/repositories and accession number(s) can be found in the article/Supplementary material.

## Ethics statement

The studies involving humans were approved by the Ethics Committee of the First Affiliated Hospital of Anhui Medical University. The studies were conducted in accordance with the local legislation and institutional requirements. Written informed consent for participation was not required from the participants or the participants' legal guardians/next of kin because the retrospective nature of the present study, the anonymity of data, and the routine nature of all clinical procedures and tests conducted, the requirement for specific informed consent was waived. Written informed consent was obtained from the individual(s) for the publication of any potentially identifiable images or data included in this article.

## Author contributions

CC: Data curation, Formal analysis, Methodology, Writing – original draft, Writing – review & editing. JJ: Data curation, Writing – review & editing. RC: Data curation, Formal analysis, Writing – original draft. YC: Data curation, Writing – original draft. KW: Supervision, Writing – original draft. ZW: Data curation, Writing – original draft. TX: Data curation, Writing – original draft. HZ: Data curation, Writing – original draft. HH: Data curation, Writing – original draft. HL: Data curation, Validation, Writing – original draft. QZ: Data curation, Validation, Writing – review & editing. XM: Data curation, Supervision, Writing – original draft. DK: Methodology, Supervision, Validation, Writing – review & editing.

## Supplementary material

Supplementary Video 1 Measurement of the EV diameter using EVR by videos. The variceal diameter of the patient was about 0.6 cm.
